# A Narrative Review on Higenamine: Pharmacological Properties and Clinical Applications

**DOI:** 10.3390/nu17061030

**Published:** 2025-03-14

**Authors:** Hanghao Shi, Long Cheng, Huixin Li, Longqi Yu, Ting You, Zhiqin Xu, Zixiang Zhou, Haotian Zhao, Chang Liu, Shengfang Shu

**Affiliations:** 1 College of Physical Education, Shanghai University of Sport, Shanghai 200438, China; shihanghao@jiangnan.edu.cn; 2 Department of Physical Education, Jiangnan University, Wuxi 214122, China; haotianzhao@jiangnan.edu.cn; 3 Russian Sports University, Moscow 105122, Russia; 4 Beijing Sport University, Beijing 100084, China; 2021011319@bsu.edu.cn (H.L.); johnny.ylq@foxmail.com (L.Y.); 15807211279@163.com (T.Y.); 2024210297@bsu.edu.cn (Z.X.); zhzx033@gmail.com (Z.Z.)

**Keywords:** antioxidant, anti-inflammatory, cardiovascular protection, PI3K/Akt signaling pathway, anti-doping

## Abstract

Background: Higenamine, a bioactive alkaloid derived from plants such as *Aconitum* and *Annona squamosa*, has been traditionally used in Chinese medicine for treating heart diseases like bradycardia, arrhythmia, and heart failure. It exhibits multiple pharmacological effects, including anti-oxidative stress, improved cellular energy metabolism, anti-apoptosis, and enhanced erectile dysfunction. Aim and Methods: To investigate the reasons for these functions of higenamine and its application in the clinic, the literature of the database was searched and read in this study. Results: As a non-selective β-agonist, higenamine activates both β_1_- and β_2_-adrenergic receptors, leading to cardiovascular benefits such as increased heart rate and myocardial contractility, as well as bronchodilation. It has also been studied for its potential in weight loss, anti-inflammatory properties, and antioxidant properties, with applications in treating asthma, cardiovascular diseases, and ischemia-reperfusion injuries. However, its clinical use is limited by the need for further research on its long-term safety, pharmacokinetics, and interactions with other drugs. Despite its promising therapeutic potential, higenamine’s inclusion in the World Anti-Doping Agency’s banned list highlights concerns over its stimulant effects and safety in athletic contexts. Conclusions: Future studies should focus on optimizing its clinical applications while ensuring safety and efficacy. In terms of clinical applications, future research will also be able to explore more possibilities to use higenamine more in the treatment of diseases.

## 1. Introduction

Higenamine is a natural compound that can be extracted from plants such as *Aconitum* (subfamilia: Chrysanthemum subfamilia, subclass: primitive perianth subclass) [1] and *Annona squamosa* (familia: Annonaceae, class: Annona) [2]. It has been used in traditional Chinese medicine to treat a variety of cardiac disorders (e.g., bradycardia, arrhythmia, heart failure). Modern studies have shown that higenamine possesses a variety of pharmacological properties, such as antioxidant properties, improvement of cellular energy metabolism, anti-apoptosis [1], and improving erectile dysfunction [3]. Higenamine acts as a non-selective β-agonist, activating both β_1_- and β_2_-adrenergic receptors [4].

Before 2015, China exhibited many clinical studies on higenamine as a pharmacological agent for the treatment of heart disease and cardiac stress testing. In 1988, Wellstein et al. compared the effects of higenamine and adrenaline on a series of mechanical parameters of the left atrium in a rabbit experiment [5]. The results showed that higenamine had a dose-dependent effect on increasing the growth rate of myocardial tone, shortening the time to reach peak tone, and shortening the total duration of contraction. As a beta-adrenergic receptor blocker, propranolol competitively blocks these effects of higenamine. In addition, the study showed that the antagonistic parameter (pA2 value) of higenamine and propranolol was 8.58 +/− 0.14, the pA2 value of epinephrine and propranolol was 7.50 +/− 0.82, and the slopes of the Schild plots of the two were 0.97 and 0.99, respectively. These results suggest that higenamine may have a positive effect on muscle strength by stimulating the adrenergic receptors of the heart [5]. In another study, it was also noted that hignamine dose-dependently relaxes guinea pig tracheal muscles. This effect was attributed to its role as a β_2_-adrenergic receptor agonist [6] and a β_1_-adrenergic receptor agonist [7]. These findings support its potential as a bronchodilator for asthma treatment.

These findings suggest that the therapeutic effects of higenamine are related to a series of pharmacological features of its structure, including bronchodilator features, anti-inflammatory properties, lipolysis, and heart rate regulation. Clinically, higenamine is used as a drug or supplement to treat asthma, lose weight, treat cardiovascular disease, and improve athletic performance [8,9]. Here, higenamine is examined in terms of its chemical structure, pharmacological effects, and clinical and athletic performance improvement. We will also summarize the signal transduction pathways involved in its action and synthesize previous studies to provide possible leads for future research.

## 2. Methods

This narrative review was subjected to a literature search in PubMed, Web of Science, and CNKI, covering the period from the inception of the database to December 2024, with the search limited to title and abstract, and the search formula including “higenamine”.

Inclusion criteria:

(1) Study design: Only randomized controlled trials (RCTs) or mechanistic pathway studies will be included. RCTs must have a clear randomization process, while mechanistic studies should focus on elucidating the biological pathways or mechanisms of action related to higenamine;
(2) Intervention: Studies must explicitly use higenamine as the primary drug or dietary supplement. This includes research investigating its pharmacological effects, therapeutic potential, or physiological impacts;
(3) Language: Studies published in English or Chinese will be considered to ensure accessibility and relevance to the target audience;
(4) Study subjects: Both animal and human studies are eligible for inclusion, provided they meet the other criteria. This allows for a comprehensive evaluation of higenamine’s effects across different biological systems.

Exclusion criteria:

(1) Study type: Studies focused solely on drug testing (e.g., doping testing methods and testing model establishment) will be excluded;
(2) Language: Studies published in languages other than English or Chinese will be excluded to maintain consistency and avoid potential translation errors;
(3) Intervention: Studies that treat higenamine as a minor component of classical drug formulation or compound mixture will be excluded. The focus is research on where higenamine is the primary agent of interest.

To be more precise, searching words include “higenamine” AND “exercise” OR “sports”, “cardiovascular”, “anti-inflammatory”, “antioxidant”, “pharmacodynamics”, “pharmacokinetics”, “chemical structure”, “pathway”, and “anti-doping” in combinations. All the studies were read after searching, and inclusion and exclusion standards were used.

The results in this paper are presented in a tabular format [10] to make them relatively intuitive.

## 3. Chemical Structure and Properties

**Figure 1:**
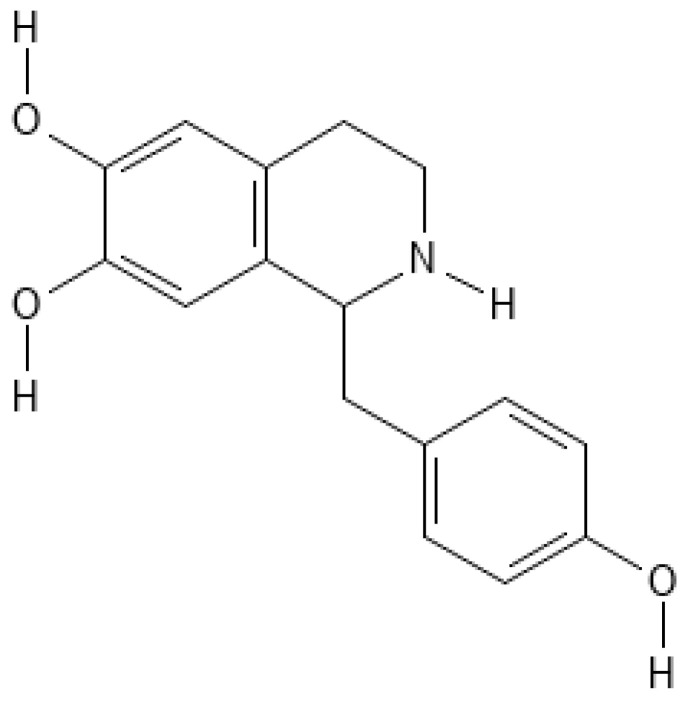
The chemical structure of higenamine.

Higenamine, classified as a phenylethylamine derivative, is an alkaloid compound that exhibits a structural resemblance to sympathomimetic agents, such as epinephrine and norepinephrine. The molecular formula of higenamine is C_16_H_17_NO_3_, and its systematic name is 4-hydroxy-3-(4-hydroxyphenyl)-N-methyl-benzenemethanamine. The compound is characterized by a benzene ring with two hydroxyl groups at the 3- and 4-positions, a methylamine functional group, and a methylene bridge connecting the aromatic ring to the amine group (Figure 1).

There are three types of adrenaline receptors in the human body, namely α_1_, α_2_, and β, each of which can be divided into three subtypes, namely α_1A_, α_1B_, α_1D_, α_2A_, α_2B_, α_2C_, β_1_, β_2_, and β_3_, a total of nine subtypes [11]. Of these, β_1_-adrenergic receptors are the most abundant in the heart, accounting for approximately 75–80% of all adrenergic receptors. β_2_- and β_3_-receptors are found in lesser numbers [11]. The negative inotropic effect of β_3_-adrenaline receptors enables them to counteract excessive cardiac stimulation by adrenaline in the heart [11,12] and has a relaxant effect on blood vessels [12]. In adipose tissue, it can cause brown fat to produce heat and white fat cells to break down [12,13]. β_1_-adrenoceptors are anti-apoptotic in the heart, while β_2_-adrenoceptors promote apoptosis [12].

### 3.1. Structural Insights and Receptor Interactions

Higenamine’s structure is primarily responsible for its ability to act as a β-adrenergic agonist [14]. In 2019, Chen et al. investigated the mechanisms underlying the therapeutic effects of traditional Chinese medicine (TCM) in treating heart failure. They focused on identifying β-adrenergic receptors as potential targets for TCM interventions and explored specific compounds that could be used for the treatment of chronic heart failure. In the study, Chen found that higenamine is a potent β-adrenergic receptor agonist. Further, in the specific inhibition experiment, the results showed that higenamine activated both β_1_- and β_2_-adrenergic receptors. They used pertussis toxin, an inhibitory G protein, as an inhibitor, and its inhibitory effect on higenamine showed that higenamine is a dual agonist of β_2_-adrenergic receptor agonist G protein and inhibitory G protein. Their research results show that higenamine is a dual agonist of β_1_/β_2_-adrenergic receptors, and there is no bias towards agonism and inhibition of G-protein stimulation in the β_2_-adrenergic receptor signaling pathway. The pharmacological role in the treatment of chronic heart failure is also discussed [15].

The authors explore which functional groups in the chemical structure of higenamine are involved in the activating β_2_-adrenergic receptor agonists, as well as the effectiveness compared with endogenous catecholamines (dopamine, norepinephrine, and epinephrine). They believe that except for the 4′-hydroxyl group, the remaining functional groups in higenamine can enhance the uptake of glucose. In addition, its ability to enhance glucose uptake is comparable to that of epinephrine and norepinephrine. Finally, the S-isomer has a stronger ability to promote glucose absorption than the R-isomer [16]. In a review, Chen et al. discussed articles related to the pharmacological effects, signaling pathways, and pharmacological targets of higenamine. Their research and reading results found that higenamine has antioxidant, anti-inflammatory, anti-apoptotic, electrophysiological regulation, and lipid-lowering effects. Because its chemical structure is like that of catecholamines, it has similar properties to adrenergic receptor ligands and can regulate targets and transcription factors related to anti-apoptosis and anti-inflammatory effects. Its combination with cucurbitacin B can enhance its anti-apoptotic activity. Chen et al. also pointed out in this review that although there has been great progress in the research on higenamine, further pharmacological research is still needed [17].

**Figure 2:**
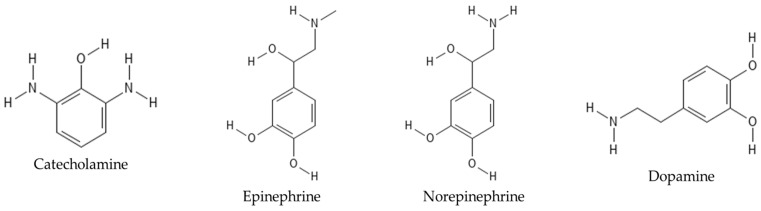
The chemical structure of catecholamine, epinephrine, norepinephrine, and dopamine.

Higenamine exerts its pharmacological effects, including bronchodilatation, vasodilatation, and increased cardiac output, primarily by acting on beta-adrenergic receptors. Adrenergic receptors are glycoproteins on the cell surface that can selectively bind to catecholamines, epinephrine, and norepinephrine secreted by sympathetic nerve endings and adrenal medulla. Their stimulation is transduced into intracellular signals, which can cause reactions including arteriolar smooth muscle contraction and cardiac contraction, maintain blood pressure homeostasis, and regulate cardiac function [18] (Figure 2).

As previously noted, Chen et al. highlighted in their study that there are mouse-based experiments demonstrating that higenamine exhibits positive inotropic and chronotropic effects on the atrium [15]. Wong et al. used the β-adrenergic receptor antagonist propranolol to block the positive induction of higenamine as a β-adrenergic receptor agonist in an isolated rat atrium. However, the relaxation of the aorta induced by higenamine was not completely antagonized by propranolol at a concentration of 1 × 10^−5^ M. In addition, its effect of inducing aortic relaxation decreased in the absence of endothelium. The results showed that the specificity of β-adrenergic receptors in the aorta for higenamine is different from that of β_1_-receptors in the atria, and most of the β-adrenergic receptors sensitive to higenamine are distributed in the endothelium [19].

Additionally, research suggests that higenamine may also interact with other receptor systems such as α-receptors and dopamine receptors, although these effects are less well understood. The exact mechanism of action, particularly in relation to its activity at β_3_-receptors, is still under investigation, as β_3_-adrenergic activation can influence lipolysis and thermogenesis, potentially enhancing fat burning and metabolic rate. For example, in a study by Lee et al., they pointed out that higenamine can stimulate lipolysis, so they conducted acute dietary supplementation of higenamine on 16 healthy subjects. They used a double-blind randomized crossover experiment. In the experiment, the subjects took higenamine, caffeine (270 mg), and yohimbe bark extract or a placebo twice. Blood was collected before and 30, 60, 120, and 180 min after each intake to measure free fatty acids and glycerol content. At the same time, metabolic-related indicators such as the respiratory exchange ratio were measured by indirect measurement, and blood pressure and heart rate were recorded throughout the process. The results showed that the values of free fatty acids were higher than those of the placebo group at 60, 120, and 180 min after taking the supplement, while the results of glycerol and the respiratory exchange ratio were not statistically significant. The results of heart rate and systolic blood pressure in the supplement group were higher than those in the placebo group. Therefore, they conclude that the results of the study on circulating free fatty acids and energy expenditure in healthy, young men and women suggest that dietary supplementation with higenamine can promote lipolysis and increase energy expenditure, with minimal contribution to increases in hemodynamic variables [20].

The above studies show that higenamine has a similar structure to endogenous catecholamines in pharmacology, which determines its function of promoting glucose absorption and utilization, as well as its anti-inflammatory and anti-apoptotic effects. These findings suggest that it could serve as a therapeutic agent or dietary supplement to modulate human metabolism and prevent the onset of diseases, particularly those associated with inflammation and aging. The potential mechanisms and pathways of action will be elaborated in the fourth part of this article.

### 3.2. Chemical Properties

Like other compounds, the chemical properties of higenamine are related to its chemical structure. It is a chiral compound containing an asymmetric carbon [21]. It is generally synthesized by Pictet–Spengler condensation of dopamine and 4-hydroxyphenylacetaldehyde under the catalysis of higenamine synthase [22]. Some people believe that higenamine is a racemic mixture of two enantiomers, namely (S)-(−)-higenamine and (R)-(+)-higenamine [21], but the higenamine isolated from plants is in the form of (R)-(+)-higenamine [22]. Current studies have shown that (S)-(−)-higenamine has good cardiotonic and anti-platelet aggregation effects [22]. Studies have shown that the anticoagulant effect of higenamine on platelets is achieved by antagonizing the induced effect of arachidonic acid on platelet aggregation by blocking thromboxane A (2) receptors [23].

These research results provide a reference for the clinical research of higenamine, provide possibilities for subsequent research on the prevention and treatment of injuries caused by cerebral ischemia–reperfusion (I/R), and point out the possible signaling pathways of higenamine in treating such injuries.

There are also many literatures discussing the possible mechanistic pathways of higenamine, which will be discussed in detail in the third part.

## 4. Pharmacological Properties

As mentioned above, higenamine exerts its pharmacological effects through its interactions with various molecular pathways, particularly by activating β-adrenergic receptors, most notably β_1_- and β_2_-receptors [15]. In an early study, it was concluded that higenamine increases the release of acetylcholine by activating β-adrenergic receptors [24]. In addition, studies have shown that higenamine can reduce oxidative damage through ROS [25,26] and exhibit anti-inflammatory activity against interleukins-1β(IL-1β)-induced inflammation by inhibiting the NF-κB signaling pathway [27]. These actions, along with its sympathomimetic properties, underpins many of its therapeutic potentials. In addition to these well-studied mechanisms, higenamine is involved in several other physiological processes that contribute to its therapeutic utility across a range of medical conditions. Below are its main pharmacological actions.

### 4.1. Pharmacokinetic Profile

In pharmacokinetic profile studies, animal experiments have shown that higenamine is rapidly absorbed after oral administration and reaches peak concentration within 10 min [28]. Feng et al. studied the pharmacodynamics, pharmacokinetics, and safety of aconitine. They selected 10 healthy subjects and injected higenamine continuously in doses gradually increasing from 0.5 μg·kg^−1^ min^−1^ to 4.0 μg·kg^−1^ min^−1^. Each dose lasted for 3 min. Blood and urine samples were collected at specific times to measure mesalamine concentrations. Heart rate was used as a measure of pharmacodynamics. Finally, plasma concentration–time curves and heart rate were modeled using a nonlinear mixed-effects model. The results showed that peak concentrations of higenamine ranged from 15.1–44.0 ng/mL with a half-life ranging 0.107–0.166 h, including a half-life of 0.133 h. Pharmacodynamic modeling: The relationship between heart rate and plasma drug concentration was determined by a simple direct effect model incorporating a baseline. The baseline heart rate (E0) was 68 bpm, the maximum heart rate was increased by bpm, and the plasma higenamine concentration at a 50% increase in maximum heart rate was 8.1 μg/L. Vons concluded that higenamine has a favorable pharmacodynamic and pharmacodynamic effect and that the results obtained may also inform subsequent clinical studies [29].

### 4.2. Cardiovascular Effects

Higenamine has been studied for its cardiovascular actions, particularly its influence on heart rate and blood pressure. As a β_1_-adrenergic agonist, higenamine acts on β_1_-receptors in the heart, leading to increased heart rate (positive chronotropy) and enhanced myocardial contractility (positive inotropy). These effects could potentially be beneficial in patients experiencing low cardiac output, such as those suffering from heart failure or hypotension.

In a rat study, it was shown that higenamine combined with 6-gingerol can treat chronic heart failure (CHF) caused by doxorubicin (DOX) treatment, which may have a protective effect on the heart. They analyzed the effects of higenamine combined with 6-gingerol on serum indexes, histopathology, and cardiac tissue hemodynamics. They measured the mitochondrial energy phenotype and mitochondrial fuel flexibility of cells and also explored the potential metabolites that affect the therapeutic effect and pathological process of higenamine. The research team also studied the mRNA and RAAS of the LKB1/AMPK/Sirt1-related pathway to explore the potential mechanism of higenamine combined with 6-gingerol. The results showed that higenamine combined with 6-gingerol can improve cardiac function, reduce serum indexes, and reduce histological damage to the heart. Higenamine combined with 6-gingerol prevents DOX-induced cardiotoxicity through cardiotonic effects and promotes myocardial mitochondrial metabolism through the LKB1/AMPK/Sirt1 pathway to prevent CHF [30] (Table 1). Studies on the treatment of DOX-induced cardiotoxicity have shown that higenamine can inhibit AMPK activation and ROS to alleviate DOX-induced cardiotoxicity, regardless of the activation of β_2_-adrenergic receptors [31]. Similarly, in the treatment of cardiomyocyte injury, many studies have demonstrated that norepinephrine can have a beneficial effect on myocardial death during acute ischemia, and that β_2_-adrenoceptor activation mediates norepinephrine’s ability to antagonize apoptosis in CMs (classical monocytes), but norepinephrine’s ability to inhibit the activation of cardiac fibroblasts (CFs) in vitro is not related to this process. TGF-β/Smad signaling and activation of CFs ameliorate pathological cardiac fibrosis and dysfunction [32].

Unlike other potent β_1_-agonists, such as isoproterenol, higenamine’s cardiovascular effects are considered relatively mild. In a Chinese study, researchers injected higenamine into dogs and measured hemodynamics, blood pressure, and heart rate. The results showed that the heart rate increased and blood pressure decreased after the injection of 5 μg/kg of higenamine [33]. This suggests that while it may have potential in treating conditions of low blood pressure or impaired heart function, its clinical application may be limited by the relatively small degree of heart rate and contractility increase. Thus, the exact role of higenamine in clinical cardiology requires further investigation to establish its therapeutic utility. In addition, the mechanism of the pharmacological effects induced by the binding of higenamine to β_1_- or β_2_-receptors needs to be further explored.

In another study, researchers injected racemic higenamine into rabbits with sinus node syndrome. Yu et al. divided 40 rabbits into two groups, normal sinus node and damaged sinus node (SND), and randomly divided each group into a treatment group and a control group [34]. The establishment of the SND model was completed by applying formaldehyde to the sinus node. The treatment group was injected with 0.04 mg of racemic higenamine per kilogram of body weight through the ear vein within 5 min. The indicators of the sinus node were measured, such as physiological function before and after medication, and the sinus node recovery time and sinus cycle length were measured. The results showed that racemic higenamine can treat arrhythmia caused by sinus node damage, and its electrophysiological effect on the sinus node function of the SND group was significantly greater than that of the normal group. It was mainly achieved by enhancing the autonomy of the sinus node and improving the sinus and atrioventricular conduction functions [34]. In addition to regulating heart rate, higenamine can also lower blood pressure. As an antagonist of α_1_-adrenaline receptors, higenamine is considered as a potential drug for the treatment of blood pressure. Studies have shown that higenamine can lower blood pressure in normotension, spontaneous hypertension, and induced hypertension models and pointed out that since it can directly bind to α_1_-adrenaline receptors, it may be a new antagonist and therefore can lower blood pressure [35].

In addition to its effects on heart rate and blood pressure, the results of an animal experiment showed that higenamine can resist the formation of blood clots [36], providing ideas for subsequent exploration of its treatment of more cardiovascular diseases.

Higenamine also showed cardioprotective effects. Dysregulation of the PI3K/Akt signaling pathway can lead to cell apoptosis, autophagy, and inflammation, which in turn causes damage to the heart from cardiotoxic drugs. Several natural compounds including higenamine (also including gingerol, apigenin, etc.) can protect the heart by regulating this pathway [37]. Zinc protoporphyrin IX (ZnPP IX) is an inhibitor of heme oxygenase-1 (HO-1), and studies have shown that the benefits of higenamine are reversed by ZnPP IX. HO-1 has significant therapeutic potential for oxidant-induced diseases such as I/R, and the results show that HO-1 plays a key role in the protective effect of higenamine on myocardial injury induced by I/R [38]. Similarly, DOX is a chemotherapy drug used to treat a variety of cancers. It exhibits chronic and acute toxicity to the heart, but the mechanism is still unclear. The researchers chose higenamine and 6-gingerol for combined treatment. The results showed that the combined treatment of higenamine and 6-gingerol can activate the PI3K/Akt signaling pathway and have a protective effect on DOX-induced cardiotoxicity [39].

In addition to its effects on heart rate and contractility, higenamine has been found to exert potential protective effects on the cardiovascular system through its anti-inflammatory and vasodilatory properties. This is of particular interest in cardiovascular disease, where inflammation plays a key role in the development of plaque formation in the arteries.

Higenamine’s vasodilatory effects, mediated through β_2_-adrenergic receptor activation, may further contribute to improving blood flow and reducing vascular resistance. By promoting vasodilation, higenamine could potentially help alleviate symptoms associated with poor blood circulation, such as angina or peripheral artery disease. Although these cardiovascular benefits are promising, additional clinical trials are necessary to determine the extent of these effects and their long-term implications for cardiovascular health.

### 4.3. Weight Loss and Lipolysis

As mentioned above, the research results of Lee et al. indicate that higenamine, as a dietary supplement, can promote fat decomposition, increase the content of free fatty acids, and improve energy consumption [20]. Many studies have pointed out that as a weight loss agent, higenamine has been widely added to weight loss products [2,40,41,42,43,44]. Thus, higenamine has the potential to promote fat catabolism and can reduce body weight as a supplement; however, the specific pathway of action requires further study.

### 4.4. Anti-Inflammatory and Antioxidant Effects and Mechanisms

#### 4.4.1. Ischemia and Reperfusion Injury Prevention

Cerebral ischemia and neuroprotection: In the rat I/R model, higenamine can improve neurological function and inhibit I/R-induced serum tumor necrosis factor α (TNF-α) and interleukins (such as IL-1 and IL-6) [45] (Table 2). As mentioned above, higenamine has anti-apoptotic and antioxidant properties. After being absorbed by the human body, it has a potential therapeutic effect in I/R injury. Based on this, Zhang et al. evaluated this therapeutic effect and studied its potential mechanism. Oxygen–glucose deprivation/reperfusion (OGD/R) causes neuronal damage, and the results showed that higenamine can improve its damage to neuronal cells. In addition, higenamine can also attenuate the induction of reactive oxygen species (ROS) and malondialdehyde production caused by OGD/R, as well as the inhibition of superoxide dismutase and glutathione peroxidase activities; it can also inhibit caspase-3 activity and Bax expression. Higenamine can increase the expression levels of HO-1 and nuclear factor erythroid 2-related factor 2 (Nrf2). These results indicate that higenamine can prevent and treat brain I/R-induced damage [46].

Possible causes: Emerging research has also highlighted the anti-inflammatory and antioxidant properties of higenamine [47], suggesting that it may play a role in the management of various chronic diseases where inflammation and oxidative stress are major contributing factors. Chronic inflammation is a key driver of many conditions, including cardiovascular disease, diabetes, neurodegenerative disorders, and even some cancers. By modulating inflammatory pathways, higenamine could potentially alleviate the symptoms and progression of these conditions.

The groups in natural phenolic antioxidants are, for example, alkoxy, carboxyl, ester, ether, and carbonyl. Xie et al. [48] pointed out that the presence of O atoms makes these phenolic antioxidants acidic or neutral (PH ≤ 7.0), while few studies have discussed alkaline phenolic antioxidants. Most of the more classic alkaloids come from plants and are often nitrogen-containing heterocyclic compounds. They can be specifically divided into 26 types, including isoquinoline, lycopodione, and quinolinepyridine. Among them, in the three types of isoquinoline, acridone, and lycopodione, the phenolic hydroxyl group can be attached to the carbon chain to form phenolic alkaloids. Through proton transfer, phenolic alkaloids can play an antioxidant role. Studies have shown that as an alkaloid, higenamine has pharmacological properties including anti-thrombotic, anti-apoptotic, and anti-inflammatory effects. These properties are related to antioxidant effects in free radical biology and medicine and can reduce the release of cytochrome C to reduce apoptotic cell death in rats with myocardial ischemia–reperfusion.

In one research paper, the authors believe that higenamine has antioxidant effects and point out that in aqueous media, it can exert its antioxidant effects through electron transfer and proton transfer, while in a lower pH environment, the ionization of H+ inhibits and weakens electron transfer. Therefore, its antioxidant activity is affected by pH [48], which provides a reference for the use of antioxidant effects of other alkaloids.

Protects the heart and brain from damage: A mouse-based study indicated that higenamine can protect cardiomyocytes from I/R injury and prevent apoptosis by activating β_2_-adrenergic receptors. It also indicated that higenamine cannot bind to β_1_-adrenergic receptors but can resist apoptosis and produce a protective effect on the heart mediated by the β_2_-AR/PI3K/Akt cascade [49].

HO-1 is a stress protein that protects the heart and brain from I/R injury. Ha et al. speculated that higenamine has benefits for hypoxic injuries such as stroke, and glial cells play a more important role than neurons in ischemic stroke. Due to their good differentiation ability and ability to respond to inflammatory factors, they selected C6 glial cells and explored whether higenamine can protect brain cells from hypoxic injury. The results showed that higenamine can upregulate HO-1 to a certain extent to protect brain cells from hypoxic injury. It can increase the expression of HO-1 in C6 cells under both normoxic and hypoxic conditions, which is more obvious in hypoxic environments. Higenamine can increase Nrf-2 luciferase activity and transfer Nrf-2 to the nucleus. It can also increase Akt phosphorylation in C6 cells [50]. It has also been demonstrated that the anti-inflammatory effects of higenamine coincide with increased expression of HO-1 [51,52] and Nrf-2, inhibition of the NF-κB signaling pathway, and reduction of the production of inflammatory factors (TNF-α, IL-6, etc.) and ROS, as well as nitric oxide (NO), among others, in microglial cells of the mouse nervous system [51]. Further, higenamine also attenuated lipopolysaccharide-induced depressive-like behavior by modulating brain-derived neurotrophic factor-mediated ER stress and autophagy [53]. It is also possible that higenamine may ameliorate and treat depression by improving astrocytes [54].

From this, we can conclude that the anti-inflammatory and antioxidant effects of higenamine in the nervous system can ameliorate the occurrence of depression or depressive-like behaviors and may provide ideas for the treatment of depression.

#### 4.4.2. Neuroprotective Effect

Higenamine significantly decreased the upregulated levels of ROS, malondialdehyde, TNF-α, and IL-6 and increased the levels of superoxide dismutase, ameliorating neuropathic pain-induced oxidative stress injury, apoptosis, and inflammation, and the neuroprotective effects might be related to the NOX2/ROS/TRP/P38 MAPK/NF-κB signaling pathway [47]. Higenamine is also neuroprotective in Alzheimer’s disease, and studies have shown that it can attenuate aluminum-induced oxidative damage by modulating oxidative markers [55].

#### 4.4.3. Anti-Inflammatory Effects

Asthma and allergic reactions: Moreover, some studies have pointed out that allergic asthma is mainly caused by Th2 cells, and the release of cytokines and inflammatory factors can lead to symptoms such as inflammatory response and airway hyperresponsiveness. In the treatment of asthma, higenamine is used as a bronchodilator. After the asthma patients were divided into two groups, one group was treated with salbutamol and the other group was treated with higenamine, the levels of immunoglobulin E, LTB4, and LTC4 were measured, and the gene expression of Muc5ac, Muc5b, Agr2, and Arg1 and histopathological studies were evaluated. The results showed that higenamine could reduce airway hyperresponsiveness, IL-4, and IL-13 levels, reduce the mRNA expression of MUC5ac, MUC5b, Arg1, and Agr2, and improve the proliferation of goblet cells and excessive mucus secretion, but it had no significant effect on IL-5, INF-γ, LTB4, and LTC4 in the lungs. Therefore, higenamine can be used to control asthma attacks and dyspnea, and has an allogeneic immune regulation effect, but the effect on inflammation and inflammatory factors is insignificant [56].

Ulcerative colitis and allergic rhinitis: In the treatment of ulcerative colitis (UC), Shao et al. demonstrated that higenamine can inhibit the Galectin-3/TLR4/NF-κB signaling pathway in UC mice [57], improve the viability and epithelial barrier function of normal human colon epithelial cells (NCM460) induced by sodium dextran sulfate, and inhibit cell apoptosis and inflammation [57]. In addition, higenamine can also be used to treat allergic rhinitis (AR), and it is believed to relieve it by activating Akt1 and inhibiting EGFR/JAK2/c-JUN signaling. This is because the results of this study showed that serum histamine, IgE, IL-4, and other levels decreased in AR mice that took higenamine orally, and Th1/Th2 cells were unbalanced. Finally, histamine-induced IL-6 and IL-8 expression and secretion levels were reduced, MUC5AC expression and NF-κB phosphorylation were inhibited, and EGFR/JAK2/c-JUN signaling was inhibited [58]. Higenamine has been shown to reduce the production of pro-inflammatory cytokines, such as TNF-α, IL-1β, and IL-6, which are involved in the pathogenesis of systemic inflammation. In addition, it may inhibit the activity of inflammatory enzymes like cyclooxygenase-2 (COX-2), a target for nonsteroidal anti-inflammatory drugs (NSAIDs). Through these mechanisms, higenamine may reduce inflammation in tissues and organs, thus protecting against tissue damage and dysfunction [27].

In addition to its anti-inflammatory effects, higenamine’s antioxidant properties further enhance its potential in combating oxidative stress. Oxidative stress occurs when there is an imbalance between the production of free radicals and the body’s ability to neutralize them with antioxidants. Excessive oxidative stress is implicated in the aging process, as well as in the pathogenesis of various chronic diseases. Higenamine may mitigate this oxidative damage by scavenging free radicals and inhibiting lipid peroxidation, which can lead to cell damage and dysfunction. These antioxidant effects make higenamine a promising candidate for the treatment of diseases associated with oxidative stress, such as neurodegenerative diseases [55] (e.g., Alzheimer’s and Parkinson’s) and cardiovascular diseases.

In summary, higenamine, as a bioactive compound with multiple pharmacological effects, exhibits potential for a wide range of applications in the areas of cardiovascular disease, weight management, and chronic inflammation. However, despite the current findings supporting its therapeutic applications, further clinical trials are needed to validate its long-term safety and efficacy to ensure that optimal results can be achieved in its application in modern medicine. Future studies should focus on the pharmacokinetic properties of higenamine, the optimal dosage, and its interactions with other drugs to optimize its clinical regimen.

### 4.5. PI3K/Akt Signaling Pathway

**Figure 3:**
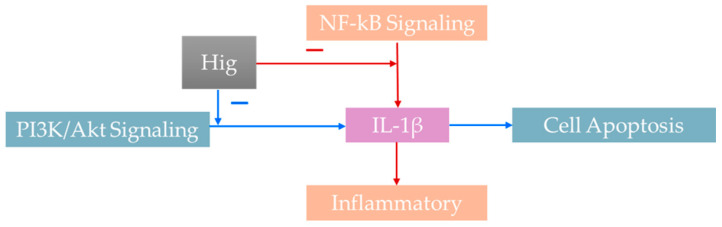
The signal pathway influenced by higenamine. (Red arrows: Standing for pathway of NF-kB influencing IL-1β, resulting inflammatory; Blue arrows: Standing for pathway of PI3K/Akt effecting IL-1β, leading to cell apoptosis. Higenamine can inhibit these two pathways).

The PI3K/Akt signaling pathway affects cell metabolism and life cycle processes such as lipid metabolism, protein synthesis, and cell proliferation and survival by mediating growth factor signal transduction. It also affects cell proliferation, differentiation, and apoptosis by blocking the activation of Akt and its downstream kinases [59]. Figure 3 shows the schematic of the signal pathway influenced by higenamine.

#### 4.5.1. Cell Apoptosis

Dysregulation of the PI3K/Akt signaling pathway induces apoptosis, autophagy, and inflammation, and drugs with these toxicities can cause damage to the heart [37]. Zhu et al. [60] discussed the effect of higenamine on IL-1β-induced apoptosis of human nucleus pulposus cells (HNPCs). In their previous study, it was proposed that higenamine could block nuclear factor-KB signaling in NPCs, thereby reducing the inflammatory response caused by IL-1β [60]. Western blotting was used to measure the proteins of the phosphoinositide 3-kinase/protein kinase B signaling pathway in the study, and it was found that higenamine had little effect on cell apoptosis but alleviated IL-1β-induced cell apoptosis in a dose-dependent manner. Higenamine can attenuate IL-1β-induced HNPCs apoptosis through the ROS-mediated PI3K/Akt pathway [60]. This study showed that higenamine can regulate inflammatory factors and thus affect the length of the cell cycle.

P53 controls cell senescence and apoptosis [61]. In the treatment of cancer, studies have found that higenamine is an effective, selective, and cell-active natural lysin-specific demethylase 1 (LSD1) inhibitor. Although higenamine has no significant effect in inhibiting the proliferation of leukemia cells, it can promote cell apoptosis and inhibit colony formation, as well as change the expression level of p53 in a way that depends on LSD1 [62]. This shows that the promotion of cell apoptosis by higenamine can provide a possible way for cancer treatment.

However, in the treatment of cancer, the combined use of higenamine and cucurbitacin B can enhance the induction of apoptosis, which may enhance the anti-tumor effect of cucurbitacin B by inhibiting the interaction between Akt and CDK2 [63]. This is the opposite of the above-mentioned combination of cucurbitacin B and higenamine to inhibit apoptosis; that is, in the treatment of tumors, the combination of the two enhances the apoptosis of tumor cells.

#### 4.5.2. Role in Diabetic Gastroparesis

There are many studies on the effects of higenamine on the PI3K/Akt pathway. Although the diseases or physiological settings studied are different, they are all instructive on the pharmacological effects of higenamine. Diabetic gastroparesis (DGP) occurs in 30–50% of diabetic patients. Some researchers believe that the depletion of Cajal interstitial cells (ICCs) is related to its pathogenesis. As pacemaker cells in the gastrointestinal tract, ICCs are very important in the process of gastric smooth muscle cells (SMCs) receiving neurotransmitters released from the presynaptic membrane. The oncogene of ICCs expresses a transmembrane tyrosine kinase receptor, namely C-kit, whose normal expression and transcription are necessary to maintain and regulate the signaling pathway of SMC contraction. G protein is a molecular switch in the signaling pathway of normal gastrointestinal contraction [64].

Gsα is a subtype of G protein. Its activation will increase the level of cAMP and lead to gastrointestinal dysfunction. Therefore, the expression levels of C-kit and Gsα are related to the occurrence of gastroparesis. Based on this, An et al. studied the treatment of gastroparesis with higenamine, and the results showed that higenamine inhibited apoptosis in the DGP rat model and maintained gastric smooth muscle cell survival by activating the β_2_-AR/PI3K/Akt pathway. The specific molecular mechanism is that the β_2_-AR/PI3K/Akt pathway plays an important regulatory role in cell proliferation and apoptosis, and higenamine plays a role in regulating cardiomyocyte apoptosis in the PI3K/Akt pathway. The results of their research on rats showed that compared with the control group, the expression of PI3K/Akt signaling proteins p-P13K and p-Akt in the model group induced by Streptozotocin (STZ, an injection that causes the DGP model in rats) was inhibited, but in the STZ + higenamine group, higenamine increased the expression of these proteins. When higenamine and STZ were added, the expressions of their downstream p-21, p-GSK3β, and p-BAD were inhibited, while all three were increased in the STZ-induced group [64].

#### 4.5.3. Treatment of Rheumatoid Arthritis

Rheumatoid arthritis (RA) is an autoimmune disease [65], in which immune system dysfunction is the main cause of RA, and the specific cause of the disease needs to be further determined.

In mammals, PI3K/Akt acts on the target of rapamycin to promote the proliferation of synoviocytes and aggravate RA [59]. Therefore, Duan et al. used the common collagen-induced arthritis animal model method to establish a model, and the induced clinical symptoms were similar to RA. After the type II collagen (CII)-induced arthritis (CIA) model was established, the researchers aimed to determine the protective effect of higenamine on collagen-induced arthritis through HO-1 and PI3K/Akt/Nrf-2 signaling pathways. They performed HO-1, Akt, and Nrf-2 protein expression in CIA mice induced by CII and detected the clinical arthritis scores, oxidative damage, and caspase-3/9 activation of mice. The results showed that higenamine significantly reduced the increase in clinical arthritis scores, inhibited inflammatory response and oxidative damage, and promoted the activation of caspase-3/9. They also performed western blot detection and analysis of HO-1 protein, and the results showed that the expression of HO-1 in CIA mice undergoing surgery was similar to that in the higenamine group, while the expression of HO-1 in CIA was significantly decreased, and higenamine significantly increased the inhibition of HO-1 protein expression in CIA mice. Finally, Duan et al. also measured the expression of Akt, p-Akt, and Nrf-2 proteins. The results showed that the expression of p-Akt/Akt protein in mice with CIA decreased, and the expression of p-Akt/Akt protein in CIA mice treated with higenamine was significantly increased; that is, the inhibition of p-Akt/Akt protein expression in CIA mice was activated. Nrf-2 expression was reduced in CIA mice, and its expression was significantly increased after higenamine treatment. Therefore, they pointed out that higenamine prevents the occurrence of CIA induced by CII by inducing HO-1 and upregulating the PI3K/Akt/Nrf-2 signaling pathway [66].

Also treating RA, Kang et al. believe that nitric oxide is a key mediator of RA. NOS2 (iNOS) mRNA and protein are transcribed and expressed in the synovial tissue of patients with inflammatory arthritis, producing nitric oxide in vitro. They wanted to explore the effect of higenamine on lipopolysaccharide-induced nitric oxide synthase. The results showed that higenamine could reduce iNOS expression in a rat aorta induced by lipopolysaccharide, and higenamine could prevent lipopolysaccharide from promoting the induction of nitric oxide synthase in vascular smooth muscle [67].

### 4.6. Treatment of Osteoporosis

In addition to the PI3K/Akt signaling pathway, studies have shown that higenamine also plays a role in preventing and treating osteoporosis. Through transforming growth factor-β (TGF-β), SMAD2/3 signal transduction plays a key role in osteogenic differentiation. Dong et al. believe that higenamine may be able to treat osteoporosis. The results of studies in mice showed that higenamine can induce the expression of osteogenic markers in mouse bone marrow stromal cells and pre-osteoblast cultures. Research data show that higenamine can promote the phosphorylation of SMAD2/3 and then regulate the SMAD2/3 pathway by inhibiting SMAD4 ubiquitin. They believe that higenamine can promote the occurrence of osteoporosis through the SMAD2/3 pathway [68].

### 4.7. Potential Health Risks

In an animal study, mice were given different doses of higenamine intravenously, and the control group was given higenamine orally. The results showed that mice receiving 60 mg/kg of higenamine intravenously died of tachycardia and respiratory distress, and that mice receiving 2 mg/kg of higenamine intravenously and mice receiving 2 mg/kg orally died of tachycardia and respiratory distress, while mice receiving 2 mg/kg of higenamine intravenously and mice receiving 2 mg/kg orally died of tachycardia and respiratory distress. Those receiving only higenamine did not die. The results of this study provide a reference for the clinical used-dose of higenamine [69]. Second, in terms of central nervous system effects, some case reports suggest that it may trigger anxiety, tremor, and insomnia, with the risk exacerbated especially when combined with other stimulants (e.g., caffeine). In addition, the toxicity of higenamine in patients with hepatic or renal insufficiency, pregnant women, or patients with cardiovascular disease is unclear due to the lack of rigorous clinical safety data. Higenamine is classified as prohibited by the World Anti-Doping Agency (WADA) not only because of its potential “performance-enhancing” effects, but also because of the potential for myocardial damage and metabolic disorders associated with its abuse. These risks are currently amplified by the lack of standardized dosages and long-term safety studies, highlighting the significant safety concerns associated with its unregulated use as a drug or supplement [70].

## 5. Clinical Applications and Anti-Doping

Higenamine has attracted increasing attention as a potential therapeutic agent for a variety of clinical conditions, particularly respiratory disorders, weight management, athletic performance enhancement, and cardiovascular diseases. Although more clinical research is needed to fully establish its safety and efficacy, preliminary studies suggest that it may provide significant therapeutic benefits in these areas. Below is an expanded overview of higenamine’s clinical applications across these different domains.

### 5.1. Respiratory Disorders

Higenamine acts primarily as a β_2_-adrenergic agonist, like conventional bronchodilators like salbutamol and terbutaline, which activate the β_2_-receptors on the smooth muscle cells of the bronchi [14,15,16,17,18,19]. By binding to these receptors, higenamine promotes the relaxation of bronchial muscles [71], leading to expanded airways and improved airflow, which is crucial for alleviating respiratory symptoms associated with asthma and COPD [72]. This mechanism of action suggests that higenamine could be beneficial in relieving bronchospasm and improving overall pulmonary function in patients with these conditions.

### 5.2. Anti-Doping

As mentioned above, many studies have pointed out that higenamine is added to weight loss products. As a stimulant, higenamine has the property of promoting fat metabolism, which can help users to increase energy expenditure and fat oxidation during weight loss, but at the same time, the stimulation of the central nervous system may harm the user, and also challenge the fairness of sports events. In 2017, it was included in the list of banned drugs by WADA (Table 3). A study pointed out that since 2014, the US Food and Drug Administration has received reports of adverse reactions caused by supplements containing higenamine, but the health risks of higenamine are still unclear [40].

Meanwhile, a three-week supplementation of higenamine in amateur female basketball players found that a supplemental dose of 75 mg did not cause weight loss or improve cardiopulmonary function in the athletes, indicating that this dose is good and non-toxic for the athletes [73]. However, since higenamine is included in the list as a banned drug, professional athletes and coaches should still be careful when using it.

**Table 3:** Several agents’ legal regulations of using higenamine.

Agents	Using Higenamine	Notes
WADA	Prohibited both in and out of competition [74]	It is listed in S3 as a β_2_-agonist in 2017 [75]
FDA	Considered an unsafe food additive in health supplements [76]	The use of higenamine has been warned [76]

Higenamine has multiple potentials for clinical applications covering its role in respiratory disorders, weight loss, and stimulant use. Higenamine has shown the ability to improve respiratory function and alleviate asthma symptoms, while its fat metabolism-promoting properties have made it a common ingredient in weight loss products. However, higenamine’s stimulant properties have also raised safety and legal concerns, particularly with regard to its banned status in sports. Although higenamine exhibits a wide range of pharmacological effects and potential clinical applications, further research is necessary to assess its long-term safety and efficacy and to ensure that it can adequately safeguard the health of users in practical applications.

## 6. Discussion

### 6.1. The Main Findings of the Review

#### 6.1.1. Clinical Applications of Higenamine

Higenamine acts as a β_2_-adrenergic receptor agonist, which can diastole bronchial smooth muscle and improve respiratory function in patients with asthma and chronic obstructive pulmonary disease (COPD). It has shown potential therapeutic benefits in the treatment of cardiovascular diseases such as arrhythmias, heart failure, and hypotension. It improves cardiac function by increasing heart rate and myocardial contractility. In competitive sports events, higenamine has been added to the list of prohibited drugs by the World Anti-Doping Agency (WADA) due to its central nervous system-stimulating effects. Despite its potential for weight loss and athletic performance enhancement, the safety of its long-term use requires further study.

#### 6.1.2. Mechanism of Action of Higenamine

Higenamine affects cell proliferation, differentiation, and apoptosis by regulating the PI3K/Akt signaling pathway. This pathway plays an important role in cardiovascular protection, anti-inflammatory processes, and anti-apoptotic processes. Higenamine exerts its cardiovascular and bronchodilatory effects through binding to β_1_- and β_2_-receptors. In addition, it may interact with α-adrenergic receptors and dopamine receptors, but the exact mechanism needs further study.

### 6.2. Strength and Limitations of the Review

This article organizes and summarizes the different perspectives of higenamine and discusses the clinical applications and possible mechanisms separately. However, the shortcomings lie in the fact that due to the limited amount of literature, it is impossible to further explain and evaluate some specific mechanisms after clinical use, and there is also an insufficient number of studies in the treatment of some diseases, so in subsequent studies, researchers can further explore its clinical application and expect more mechanism discoveries or doctrines.

### 6.3. The Clinical Implication of Findings

Higenamine can activate β_1_- and β_2_-receptors, thereby increasing heart rate and myocardial contractility, and has cardiovascular protective effects. Studies have shown that higenamine improves cardiac function, reduces myocardial injury, and exerts a protective effect in ischemia–reperfusion injury. Speaking of the anti-inflammatory and antioxidant effects, higenamine exhibits significant anti-inflammatory and antioxidant effects through inhibition of the NF-κB signaling pathway and activation of the Nrf2/HO-1 signaling pathway. These effects make it potentially useful in the treatment of inflammatory diseases (e.g., asthma, ulcerative colitis) and oxidative stress-related diseases (e.g., neurodegenerative diseases). Higenamine is widely used in weight loss products because of its ability to promote lipolysis, increase free fatty acid content, and improve energy expenditure. Showing neuroprotective effects in cerebral ischemia/reperfusion injury, it can reduce apoptosis and inflammatory response of nerve cells and improve nerve function.

### 6.4. The New Direction for Future Research

Although higenamine has shown potential therapeutic benefits in several areas, its safety and pharmacokinetic properties for long-term use require further study. Higenamine’s interactions with other drugs and its dosage and regimen in the clinic need to be further optimized. Future studies could explore the use of higenamine in the treatment of additional diseases, such as neurodegenerative diseases, cancer, and metabolic diseases.

## 7. Conclusions

Higenamine is a versatile and bioactive alkaloid with significant pharmacological properties, including bronchodilation, weight loss facilitation, and cardiovascular protection. Although current evidence supports its potential therapeutic applications, the safety of long-term use remains uncertain due to limited clinical trials, and its interaction with other medications requires further investigation. Despite these challenges, higenamine shows promise as a valuable therapeutic agent across several medical domains. Rigorous clinical trials, improved regulation, and comprehensive safety assessments are essential for determining its place in modern medicine and ensuring its safe use in various patient populations. With proper investigation, higenamine could become an important addition to the pharmacological arsenal for treating respiratory and cardiovascular diseases, as well as aiding weight management and athletic performance.

## Figures and Tables

**Table 1:** The summary of studies on higenamine’s cardiovascular effects.

First Author	Experimental Subjects	Outcomes	Results	Treating Disease/Unhealthy Symptoms
Wen, J. [30]	Rats	Effects on cardiac functions, energy metabolism and related pathways, mitochondrial respiratory function	Higenamine combined with 6-gingerol can improve cardiac function, reduce serum indexes, and reduce histological damage to the heart. Combining with 6-gingerol prevents DOX-induced cardiotoxicity through cardiotonic effects and promotes myocardial mitochondrial metabolism through the LKB1/AMPK/Sirt1 pathway to prevent CHF.	DOX-induced chronic heart failure
Jin, C. [31]	Rats	Assessment of reactive oxygen species (ROS) levels, superoxide dismutase levels	Higenamine alleviates DOX-induced chronic myocardial injury by inhibiting AMPK activation and ROS production.	Cardiotoxicity is caused by DOX
Zhu, J. [32]	Rats	Heart size, cardiac fibrosis, heart function	Higenamine has a beneficial effect on myocardial death during acute ischemia. It may act through TGF-β/Smad signaling and activation of CFs which ameliorate pathological cardiac fibrosis and dysfunction.	Effect of HG on chronic cardiac remodeling
Huang, N. [33]	Dogs	Blood pressure, heart rate	The heart rate increased and blood pressure decreased after the injection of 5 μg/kg of higenamine.	Effects on cardiovascular system, higenamine acts matched group
Yu, F. [34]	Rabbits	Sinus node recovery time, corrected sinus node recovery time, total sinoatrial conduction time, sinus cycle length	Higenamine can treat arrhythmia caused by sinus node damage, enhance the autonomy of the sinus node, and improve the sinus and atrioventricular conduction functions.	The mechanism of racemic higenamine in treating sick sinus syndrome
Zhang, N. [35]	Rats	Effects on arterial pressure, higenamine binding for α_1_-adrenergic receptor, and so on	Higenamine can lower blood pressure in normotension, spontaneous hypertension, and induced hypertension models.	Whether higenamine can act as an α_1_-adrenoceptor antagonist in affecting blood pressure
Yun-Choi, H.S. [36]	Rats	Anti-thrombotic activities of higenamine, protection of mice from thrombotic challenge	The results showed that higenamine can resist the formation of thrombotic effects.	Higenamine’s anti-platelet and anti-thrombotic effects
Yarmohammadi, F. [37]	-	-	Higenamine can protect the heart by regulating the PI3K/Akt signaling pathway.	Higenamine’s role in regulating PI3K/Akt pathway to protect cardiotoxicity caused by drugs
Lee, Y.S. [38]	Rats	Caspase-3 activity, heme oxygenase (HO) enzyme activity, and so on	Higenamine can trigger anti-apoptosis induced by zinc protoporphyrin IX (ZnPP IX), and HO protects beneficial effects produced by higenamine.	Higenamine’s effects on myocardial I/R-induced injury
Chen, Y.L. [39]	Rat cardiomyocytes (newborn)	Cytochrome c releasement from mitochondria, cell viability assay in H9c2 cell and neonatal rat cardiomyocytes	Combined treatment of higenamine and 6-gingerol can activate the PI3K/Akt signaling pathway and have a protective effect on DOX-induced cardiotoxicity.	Heart failure caused by DOX

**Table 2:** Higenamine’s effects on ischemia and reperfusion injury prevention.

First Author	Experimental Subjects	Outcomes	Results	Treating Disease/Unhealthy Symptoms
Wang, X. [45]	Rats	Neurological function, TNF-α, interleukins (ILs)	Higenamine can improve neurological function and inhibit I/R-induced serum (TNF-α) and ILs (such as IL-1 and IL-6).	In the I/R model
Zhang, Y. [46]	Rats	Oxygen–glucose deprivation/reperfusion (OGD/R)-induced cell injury in neuronal cells, OGD/R-induced oxidative stress, Akt and Nrf2/HO-1 signaling pathways	Higenamine can prevent and treat brain I/R-induced damage.	Test higenamine’s acts in OGD/R-induced neuronal cells injury
Yang, B. [47]	Rats	The level of ROS, glutathione (GSH), superoxide dismutase (SOD), THF-α, IL-6	Higenamine may play a role in chronic diseases related to inflammatory and oxidative stress.	The possible mechanism of higenamine in treating neuropathic pain
Xie, Y.L. [48]	-	Antioxidant value	Higenamine is a powerful antioxidant.	Antioxidant activity study of higenamine
Wu, M.P. [49]	Rats	Level of caspase-3, Akt, and pAkt	Higenamine’s anti-apoptosis and myocardium-protecting function is related to the β_2_-AR/PI3K/Akt pathway.	The molecular target and mechanism responsible for the effect of higenamine in cardioprotection
Ha, Y.M. [50]	The experiment was based on C6 cells	Nrf-2 transfection, signal pathway of induction of HO-1 by higenamine	Higenamine can increase Nrf-2 luciferase activity and transfer Nrf-2 to the nucleus.	Test higenamine’s benefits in hypoxic injuries such as stroke
Yang, S. [51]	Rats	The level of TNF-α, IL-6, ROS, NO mediated by iNOS, PGE_2_ mediated by COX2	Higenamine achieves its anti-inflammatory and antioxidant effects by inhibiting NF-κB and activating Nrf2/HO-1 signaling pathway expression.	Explore the neuroprotection effects of higenamine in neuronal inflammation
Zhang, Z. [52]	Rats	Adoptive transfer of HG-treated macrophages, factors of immunohistochemistry	Higenamine increases the expression of IL-4 and IL-10, promoting the rise in HO-1.	The effects of higenamine on spinal cord injury
Chen, S. [53]	Rats	NO production, immunohistochemistry	Higenamine attenuates LPS-induced depressive-like behavior by modulating brain-derived neurotrophic factor (BDNF)-mediated ER stress and autophagy.	Explore the molecular mechanism of depression-like behaviors
Yao, J. [54]	Rats	Changes in astrocyte GJs function, expression and phosphorylation of connexin 43	It is possible that higenamine may ameliorate and treat depression by improving astrocytes.	The potential of higenamine in treating neurodisease
